# Neural Correlates of Music Listening: Does the Music Matter?

**DOI:** 10.3390/brainsci11121553

**Published:** 2021-11-24

**Authors:** Mark Reybrouck, Peter Vuust, Elvira Brattico

**Affiliations:** 1 Faculty of Arts, University of Leuven, 3000 Leuven, Belgium; 2 Department of Art History, Musicology and Theater Studies, IPEM Institute for Psychoacoustics and Electronic Music, 9000 Ghent, Belgium; 3 Center for Music in the Brain, Department of Clinical Medicine, Aarhus University, 8000 Aarhus, Denmark; petervuust@gmail.com (P.V.); elvira.brattico@clin.au.dk (E.B.); 4 The Royal Academy of Music Aarhus/Aalborg, 8000 Aarhus, Denmark; 5 Department of Education, Psychology, Communication, University of Bari Aldo Moro, 70122 Bari, Italy

**Keywords:** neuroaesthetics, musical-aesthetic experience, allostatic load, homeostatic regulation, reward circuit, hedonic pleasure, eudaimonic experience, hypothalamic-pituitary-adrenal axis, chills and thrills, arousal

## Abstract

The last decades have seen a proliferation of music and brain studies, with a major focus on plastic changes as the outcome of continuous and prolonged engagement with music. Thanks to the advent of neuroaesthetics, research on music cognition has broadened its scope by considering the multifarious phenomenon of listening in all its forms, including incidental listening up to the skillful attentive listening of experts, and all its possible effects. These latter range from objective and sensorial effects directly linked to the acoustic features of the music to the subjectively affective and even transformational effects for the listener. Of special importance is the finding that neural activity in the reward circuit of the brain is a key component of a conscious listening experience. We propose that the connection between music and the reward system makes music listening a gate towards not only hedonia but also eudaimonia, namely a life well lived, full of meaning that aims at realizing one’s own “daimon” or true nature. It is argued, further, that music listening, even when conceptualized in this aesthetic and eudaimonic framework, remains a learnable skill that changes the way brain structures respond to sounds and how they interact with each other.

## 1. Introduction

Music listening is an experience that has been operationalized by psychologists and neuroscientists in recent decades as mainly concerning the auditory system and cognitive and affective functions. Research has for the most part focused on the perceptual and cognitive consequences of listening, linking it with the phenomenon of neuroplasticity or neural changes deriving from adaptation to new environmental demands as in the case of sensory deprivation or repeated exercise (see [1,2,3] for an overview). Consequently, music and musicians have been identified as a promising model for neuroplasticity [4], as the result of intense repeated exercise focused on the improvement of domain-specific skills. These neuroplastic changes, however, are not limited to the yearlong physical acts of motor practice with an instrument, as in the case of professional musicians. Merely listening to music in the course of days, or even hours and minutes, can result in modifications of the functioning of the brain [1,2,3,5]. In the context of the predictive coding theory of music, music listening is an active process of prediction and updating those predictions, which occurs at any exposure to new sounds over all temporal courses, from seconds up to an entire life [6,7]. This never-ending process is well exemplified by the famous cellist Pablo Casals who, when asked why he continued to practice at age 90, replied “Because I think I’m making progress”.

Listening to music, by definition, refers to the sensorial act of processing acoustic features by the auditory system. Hence, a description in terms of the objective acoustic characteristics may help to tackle some of the elusive aspects of possible causal relationships between music and its effects by describing at least the stimulus side of the music processing chain (input). Using neural correlates of stimulus processing could do the same for measuring also objectively the responses of each individual listener (output). For this, psychoacoustic research and computational developments are essential. Music listening, though, does not restrict itself to the sensorial act of processing objective features of sounds that act as triggers for passive recipients. It may be described also as an *aesthetic experience*, which, when referring specifically to music listening, is defined by Brattico & Pearce as “[an experience] in which the individual immerses herself in the music, dedicating her attention to perceptual, cognitive, and affective interpretation based on the formal properties of the perceptual experience” [8] (p. 49). Following this view, listening can be conceived as a sequence of “states” induced by active engagement with the music and by processes which can be labeled as “coping“ with the sounds [9,10]. Such definition of a musical aesthetic experience is strongly founded on empirical research but it is also in line with previous philosophical/phenomenological accounts aiming to describe the nature of the human relation to art in general as a subjective experience. Ingarden pointed to the role of the subject-driven experience, as a teleological experience to be instantiated in the concept of an encounter between the artist/performer and observer, holding a dynamic tension between the artwork as an ultimate, finished and fixed source (Ergon) and the experience as an individual process of apprehending and knowing (Energeia) [11,12] (see also [13]). Dewey, on the other hand, revoked the idea of the aesthetic experience as a question of mere knowledge and moved gradually in the direction of humans craving meaning in the sense of lived, embodied experiences with full involvement with the world. An experience, for Dewey, is a synonym for consummatory experience with the felt underlying quality of emotionally pervading the whole and giving it a sense of closure or resolution [14,15]. Mead stated that aesthetic experiences enhance the power to catch the enjoyment of the actual consummation of that experience; they are the basis for the development of self-consciousness through subjective sensations, emotions, and imagination, and are even necessary for understanding social life [16]. Dufrenne, one of the most distinguished researchers on the relationship among art, aesthetics, and phenomenology, presented three distinct categories of aesthetic perception—presence, representation and feeling—, thus providing a vast description of the essential aspects of the aesthetic experience with the aim to understand them also within the totality of the human experience [17,18]. 

Besides these philosophical definitions of the aesthetic experience, more operational definitions conceived in relation to visual art and stemming directly from empirical research were proposed by Marković, Pelowski, and Leder: according to them, the aesthetic experience brings together the biological basis of aesthetic preference and attraction and the neuroscientific study of awareness and special states of consciousness [19,20,21]. In line with this, our approach focused on the musical aesthetic experience identifies three major outcomes of aesthetic music listening: the experience of aesthetic emotions, such as, e.g., enjoyment, chills, nostalgia, awe, and being moved; aesthetic judgments as in the conscious evaluation of the beauty of the music, based on its formal properties; and a verdict of liking or preference, involving both the conscious liking/disliking of the music [8]. 

Intermediary processes between input and output, further, can substantially bias and modulate the final aesthetic responses to sounds. Among those, *personality factors* may have a critical role in eliciting emotional and physiological reactions, such as the propensity to experience aesthetic chills and thrills [22], though it can be questioned to which extent these personality characteristics act as dispositional traits that function as causal factors for the elicitation of a typical response. All these processes and modulating factors, finally, constitute a genuine aesthetic listening situation [23,24,25]. 

In sum, new research within the neuroaesthetic framework, besides focusing on perceptual and cognitive aspects of music listening in relation to prolonged sound exposure and practice in musicians, reaches out to capture the vast complexity of the music listening phenomenon. Here, we aim to provide a narrative overview of the recent research on music listening, with an effort to provide the latest organized and systematically structured knowledge on music listening as an aesthetic experience. For doing so, we will answer the following questions: (1) how does listening produce effects that go beyond the auditory system? (2) what is the relation between the style of music, its life-long exposure time, and the resultant effects on brain activity? (3) what is the role of the individual differences between listeners with their personality traits and individual listening history? and, (4) can modes of listening and strategies be reduced to their corresponding neural correlates? Our final goal is to determine whether musical styles and listening modes have an effect, both in the real-time situation of actual listening and by considering the lasting effects on the brain, and on allostatic loads and homeostatic regulation (see below for definitions of the terms).

## 2. Does the Music Matter? Acoustic Features as Candidate Elicitors of Induced Responses

There are different tonal, harmonic, and rhythmical-metrical systems and innumerable kinds of musical instruments around the world. Most of them can be related to basic principles of psychophysical and neurobiological processing of the sounds. The physical description of sound, further, is amenable to acoustic analysis and specific acoustic cues. It highlights the special importance of manipulations of acoustic frequency—as a central element of music-making [26] and timbre-related characteristics, such as the attack time, the spectral centroid, and the spectral flux [27]. Several acoustic features that capture timbral, rhythmical and tonal properties have been investigated in this regard, relying mainly on techniques from Music Information Retrieval (MIR). 

A compilation of such features in the Matlab environment derived from psychoacoustic and MIR research is the *MIRToolbox* [28], which provides a feature set that can be classified into short- and long-term features. Short term features—defined as being within a 25 ms analysis window—include timbral properties such as zero crossing rate, spectral centroid, high energy-low energy ratio, spectral spread, spectral roll-off, spectral entropy, spectral flatness, roughness, RMS energy, spectral flux, and Sub-Band Flux; the latter include pulse clarity, fluctuation centroid, fluctuation entropy, musical mode, and key clarity. All these features are related to one of the traditional dimensions in music theory, such as pitch and tonality, rhythm, timbre, and dynamics (see Box 1 for clarification). Further reduction of the dimensionality of the features set by means of Principal Component Analysis has led to six components categorized as such and validated after listening tests: Fullness, Brightness, Activity, Timbral Complexity, Pulse Clarity, and Key Clarity [29,30].

Research combining MIR with music listening and brain measurements has identified several interesting mechanisms. First, an association between electrophysiological brain oscillations at narrow frequency bands and the time course of acoustic features has been identified and replicated. Indeed, rapid acoustical changes in real music—such as changes in brightness, root mean square (RMS) amplitude, zero-crossing rate, and spectral flux (see [31] for technical details)—induce transient evoked cortical responses [31,32], similar to the ones observed in relation to isolated simple sound feature changes, such as a new frequency, or timbre, or duration, or even contour, and meter [33,34,35,36]. Moreover, both in the peripheral and central auditory system magnitudes in acoustical features, such as, e.g., their sound intensity, are linearly related to evoked response amplitudes. Similarly, the repetition of a specific feature parameter is encoded and learned by the central auditory system. The linear-causal relation between auditory features and neurophysiological responses of the central auditory system can be quantified using electroencephalography (EEG). This holds for both the decrease of the main feature-dependent auditory-cortex response according to the amount of feature repetition (N1) and the increase of prediction error signals, namely the mismatch negativity (MMN) linked to the deviation of the error from the repetitive sound context.

Other studies combining music, MIR, and brain methods that utilized functional magnetic resonance imaging (fMRI) demonstrated that music listening recruits and connects large-scale brain networks of cerebral cortical, subcortical, and cerebellar cortical regions involved with audition, motor imagery, and planning as well as emotion [29,37,38,39].

Box 1 **Overview of the feature set of the MIRToolbox for acoustic information retrieval [28,30] and their Principal Component Analysis dimensional reduction [29] (see also [40]).**

 
**TIMBRAL FEATURES**
 ***Zero Crossing rate*** (ZCR): number of time-domain zero crossings or sign-changes of the signal per unit of time
 ***Spectral centroid***: geometric center on the frequency scale of the amplitude spectrum, indicating the average amount of the energy
 ***High-energy-low energy ratio***: ratio of energy content below and above 1500 Hz.
 ***Spectral entropy***: quantifies the spectral complexity and irregularity of energy distribution in the frequency domain
 ***Spectral roll-off***: the frequency below which a specified percentage (cutoff) of the total spectral energy exists (85% by default)
 ***Spectral flux***: dynamic variation of spectral information by measuring how quickly the power spectrum of a signal is changing
 ***Spectral spread***: standard deviation of a spectrum
 ***Spectral flatness***: tonality coefficient or Wiener entropy of a spectrum that qualifies how tone-like a sound is as opposed to being noise-like
 ***Sub-Band Flux***: measure of fluctuation of frequency content (prominent partials) in different sub-bands of the spectrum
 ***Roughness***: estimate of sensory dissonance
 
**LOUDNESS**
 ***Root Mean Square Energy*** (RMS): measure of instantaneous energy contained in the signal, obtained by taking the square root of sum of the squares of the amplitude
 
**TONAL FEATURES**
 ***Mode***: strength of major or minor mode
 ***Key clarity***: measure of tonal clarity
 
**RHYTHMIC FEATURES**
 ***Fluctuation centroid***: geometric mean of the fluctuation spectrum that represents the global repartition of rhythm periodicities within the range of 0–10 Hz, indicating the average frequency of these periodicities
 ***Fluctuation entropy***: Shannon entropy of the fluctuation spectrum that represents the global repartition of rhythm periodicities, measuring the noisiness of the fluctuation spectrum.
 ***Pulse clarity***: estimate of clarity of the pulse

**Figure 1:**
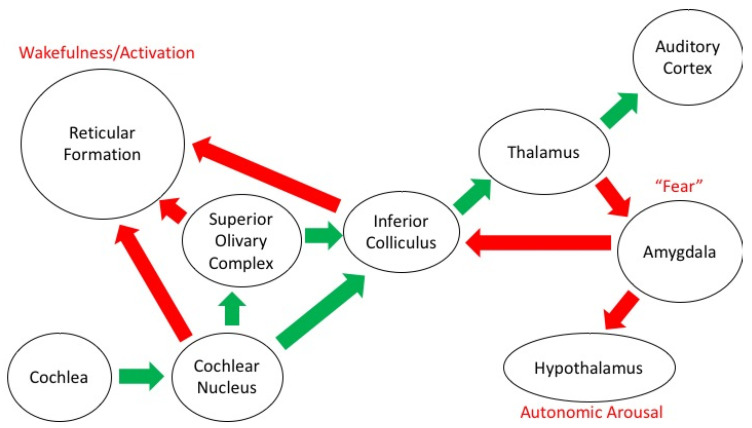
Schematic diagram of the auditory projections in the brain. The classical auditory pathway that conveys information about sound from the ear to the cortex is shown in green, the other projections to structures related to emotion and arousal are shown in red (Figure reproduced without modification from [43]. (Copyright © 2019 Reybrouck, Podlipniak and Welch. Creative Commons Attribution License (CC BY))

There are, in this regard, two auditory pathways in the central nervous system: the classical pathways from the inner ear to the auditory cortex, and pathways to the reticular activating system, with connections to the limbic system and the autonomic nervous system (see Figure 1). The pituitary-adrenal neuroendocrine system is involved in the secretion of corticosteroids, which have a major role in the management of stress through the sympathetic-adrenal system that mediates the secretion of catecholamine, adrenaline, and noradrenaline [41,42].

Music listening, further, is not simply a causal input-output chain, with stimuli leading to predictable perceptual and cognitive responses in the listener [44]. Along with the effects of music as described in the previous section, music listening causes affective and physiological reactions, linked to an aesthetic experience. The most studied ones are thrills (see [45,46,47,48,49,50] for an overview), which are considered the most common and least differentiated aesthetic responses of the “aesthetic trinity,” which besides the thrills, also includes aesthetic awe and the state of being moved [51,52]. They have been described both in terms of objective bodily processes and subjective bodily sensations, either as warm chills, when characterized by terms as warmth, smiling, happiness, stimulated and relaxed (warm chills), cold chills with terms as coldness, frowning, sadness and anger and moving chills with terms as a lump in the throat, tears, affection, tenderness, being moved and intensity [53]. 

Much can be learned, regarding musical experiences, from research on arousal and valence perception in emotional vocalizations across diverse animal classes and human beings, as prototypical examples of modulation of specific acoustic parameters. The link with music may seem obvious—at least for certain kinds of music—but systematic research on this topic is still waiting for additional research. Much is to be expected here from the broader domain of acoustic roughness and typical applications in human and animal screams.

**Figure 2:**
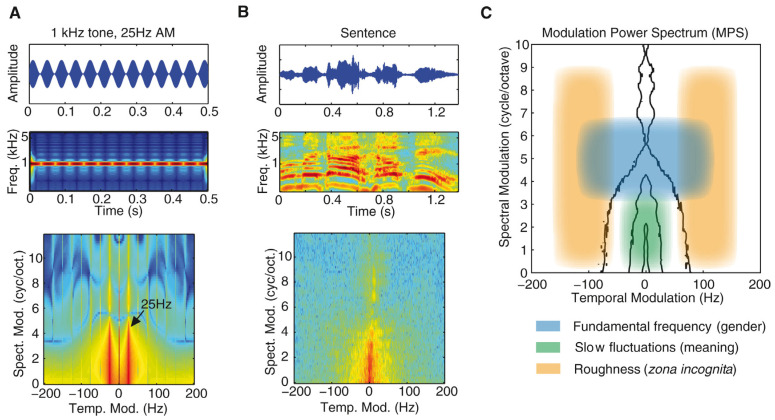
Representations of a 1000 Hz tone amplitude modulated at 25 Hz (**A**) and a spoken sentence (**B**) with waveform (top), spectrogram (middle) and MPS power modulations in the spectral (y axis) and temporal (x axis) domains (bottom). Modulations in human vocal communication (**C**) show how perceptual attributes occupy distinct areas of the MPS and encode distinct categories of information: modulations corresponding to pitch (blue) carry gender/size information; temporal modulations below 20 Hz (green) encode linguistic meaning; and orange rectangles delimit roughness (Figure reproduced without modification from [59]. (Copyright © Elsevier 2015, Licence number 5185310806708))

The ultimate aim of the acoustic modulations in these typical vocalizations is to facilitate correct identification of heightened arousal and emotional content, to make it possible to perceive potential levels of threat or danger and to react adaptively. It is a process that appears to be dominant over verbal content, seen from an evolutionary point of view [54]. Among such emotional vocalizations in human beings screaming, has received a lot of scholarly interest, due to its quasi-universal practical relevance as one of the most important alarm signals for survival. The acoustical features are well-documented either through waveform and spectrogram representations and more recently also by using modulation power spectrum (MPS), as a useful tool that provides a neurally and ecologically relevant parameterization of sounds by displaying the frequencies of temporal and spectral modulations in the spectrogram (see Figure 2). It can be defined as a two-dimensional Fast Fourier Transform of a spectrogram that shows the irregularity of a sound and the power of its temporal and spectral modulations. As such, it provides a fingerprint of the sound, which is a much more effective identifier than mere pitch or loudness representation (see [55] for technical details). 

MPS descriptions of human screams show that they cluster within a restricted portion of the acoustic space with a modulation rate between 30 and 150 Hz. [55,56,57]. They correspond to the perceptual attribute of roughness, which is a typical feature of both natural and artificial alarm signals (e.g., buzzers, horns), with the aim to boost detection by occupying a privileged acoustic niche that is easily segregated from other signals. Musical instruments, on the other hand, are characterized by much more complex spectrotemporal features, especially when they operate within the roughness range. Acoustic roughness and screams, further, activate the primary auditory cortex and the amygdalae. These structures are linked to the neural coding mechanisms that enable quick responses to acoustic cues, which are related to auditory salience and potential danger and so are critical for a rapid appraisal to ensure biological efficiency [58]. Other features that modulate or accentuate speech and/vocalizations are increased loudness and high pitch, but although they contribute to potential fear responses, they are not sufficiently distinctive to evoke the specific involved emotions. 

In this regard, it has been hypothesized that arousal-related universals may be shared also by music [60] with specific acoustic cues that seem to affect the ratings of arousal. Examples are the fundamental frequency (F_0_), harmonics-to-noise ratio (HNR), the spectral center of gravity (SCG), and duration [61,62,63]. They may be considered major underlying mechanisms for the affective outcomes of musical stimuli. Indeed, distinct MPS parameter values characterize consonant and dissonant musical intervals, with dissonant intervals generating stronger modulations in the lower half of the roughness window (30 to 80 Hz) to elicit temporal modulations that are exploited to communicate danger [59]. 

A distinction can be made between those elementary perceptual features of the sounds that produce hedonic sensations by themselves and those that are perceived as being threatening or potentially harmful. There is, in fact, a major difference between so-called relaxing, calming, and soothing music or sounds and stimulating or exciting music. Play-songs and lullabies are typical examples of the first [64] but the findings for relaxing classical music, in general, are not yet conclusive at this moment [65]. Techno music, on the other hand, has been found to belong to the second category of arousing signals [66]. Also, certain styles of rock music, with human screams that resemble natural alarm signals, are characterized by high roughness [58]. In many cases the aesthetics of pop, rock, techno, and metal—sometimes referred to as the “sound-as-power approach—are also concerned about the distribution, balance and dynamics of spectral energy to create a saturated, dense sound [40,67,68].

The impact of these acoustic features is evident even when listening to music without any attentional resources, such as when music is heard in the background while listeners are concentrating on other primary tasks. This type of listening is termed *incidental* and is by default ongoing even during early sleep stages (when most external stimulation processing is hindered and filtered out), as an adaptive way of monitoring the environment for potential dangers [69].

Some of the above-described acoustic features are more compelling than others—with low-level features being processed in a quasi-automatic way—, and even high-level emotional and cognitive processes are not functioning independently from the acoustical characteristics of the music. It might be tempting, therefore, to conceive of the relationship between the acoustic features of the music and the evoked responses in the listener in terms of a mathematical function, with as the domain all possible input values, as the range of all possible outcomes, and claiming a unique relationship or one-to-one mapping between input and output as the transfer function. Such a causal-linear relationship, however, has proven to be somewhat illusionary. Besides the universal psychophysical commonalities at the lowest levels of perception, there are many intermediary modulating factors—including intrapersonal, interpersonal, and external factors—, which may influence the actual outcome of the listening process [1,70]. Yet, it remains tempting to conceive of music listening in terms of the axiom of *psychobiological equivalence*, which addresses the central question of whether there is some lawfulness in the coordination between sounding stimuli and the response of music listeners in general [71]. It invites us to argue for an operational approach, which contains three elements: an objective description of the acoustic features of the music and their possible role as elicitors, a description of the possible modulating factors—both external/exogenous and internal/endogenous ones—, and a continuous and real-time description of the responses by the listener, both in terms of their psychological reactions and their physiological correlates.

## 3. From Incidental Listening to Full-Fledged Aesthetic Experience: The Role of Individual Factors

Responses to sound features can vary widely among individuals, depending on several modulating factors. Among them, the most studied that relate to person-specific characteristics or “internal context” are expertise, internal state, mood, personality, and attitude. The factors that relate to the listening situation, also described as “external context” are the physical and social environment, specifically whether being in a concert hall or at home, or whether being alone or with others [70,72]. Hence, when analyzing the different ways those factors affect music listening, there exist considerable differences in the ways listeners deal with music, ranging from incidental listening to an attentive skillful situation up to a life-changing aesthetic experience. We will herewith review evidence on the different types of person- and context-dependent listening experiences.

Based on the literature (see [1,70] for a broad overview) it is possible to identify specific factors that determine the individual differences in how listening occurs. They relate to innate characteristics and ontogenetic development, combining innate disposition with natural maturation and external mediation. There is no space to go into detail here, but much of the “individual difference literature” on personality and cognitive traits has drawn on the Big 5 personality traits—Extraversion, Agreeableness, Conscientiousness, Emotional Stability/Neuroticism, and Openness to experience [73]—and the role of trait empathy. Underlying personality traits have been found to mediate physiological and psychological reactions to different styles of music [22,48,65,74,75]. Empathy, on the other hand, is another factor that shapes the musical experience. It consists of two distinctive elements, coined as the affective and cognitive element, which can trigger emotional reactions, which are evoked by observed emotions of others (affective empathy) or entail a cognitive recognition or understanding without necessarily experiencing them (cognitive empathy) [76,77,78,79,80,81,82,83].

As to the ontogenetic development, accumulating exposure to sounds is the first factor at play: listeners who can draw on a lifetime’s experience can make sense of music in ways that children or teenagers cannot. There is, in this regard, the importance of each individual learning history, which may be driven by internal or external forces such as innate curiosity, the tendency to invest in exploratory behavior, and the urge to master (intrinsic motivation) or to comply with the demands of parents and/or educational institutions (external motivation). The cumulative effect of these forces may result in levels of sophistication that outreach those of simple natural maturation. 

Directly in relation to the individual lifetime experience with sounds, one way of listening is what could be termed *skillful***,** namely the listening skills acquired by exposure towards achieving expertise. While motor advances in “skill acquisition” of professional musicians, who learn fine motoric abilities specific to their instrument, are obvious in executive expert behavior, as in virtuoso performing, advances of experts are less obvious in internalized forms of behavior, such as listening to music. Musicians, though, are better at psychoacoustic processing. They show better pitch acuity than nonmusicians, and a better capacity to understand speech in a noisy environment or to extract the prosody contour of speech [84]. In terms of brain mechanisms that underlie skillful listening in music experts, meta-analyses demonstrate an enlarged volume of the gray matter for primary and non-primary auditory regions in the temporal lobes [85]. Neurophysiological measures also demonstrate the faster and stronger synchronization of neuronal assemblies when responding to sounds and to errors of sounds in musicians as opposed to nonmusicians.

Related to such skillful listening is *cognitive mastering,* a crucial stage of information processing that leads to the aesthetic outcomes of judgments and emotions as the outcome of knowledge and understanding [86,87]. Professional musicians, e.g., have access to different cognitive strategies and auxiliary representations of music in comparison with musical laymen. They show auxiliary mental representations of music as the result of training and practice and use larger and more complex neuronal networks than non-professionals with, among others, left hemisphere activation, attributed to the recruitment of inner speech by naming pitches and harmonies more or less automatically while listening. This points in the direction of brain substrates of music processing that are the outcome of ways of listening rather than being amenable to fixed music centers [88]. 

A further step of the journey into the music listening experience occurs with an intensification of the engagement with the sounds. It is somewhat related to the transition from mere *hedonic pleasure* to *eudaimonic experience*, with a shift from mere reactivity to auditory stimuli to a full-fledged aesthetic listening experience. Hedonic pleasure aims at pleasure or personal happiness, with a focus on the experience of pleasant feelings and a balance between positive and negative affect; the eudaimonic experience, on the contrary, is inspired by Aristotle’s instigation to realize one’s own “daimon” or true nature and has broader goals such as the realization of the potentials and mechanisms through which we achieve personal growth to make meaning and seek performances in our life [89,90,91]. Neuroimaging studies confirm the qualitative nature of the differences between incidental or even skillful listening and aesthetic listening, with a gradually enhanced functional connectivity between important perceptual-cognitive, attentional and reward networks of the brain and more in particular between mesolimbic and orbitofrontal reward circuits, the auditory cortex and the prefrontal cortex ([1,2], for a review of findings also related to the aesthetic experience of figurative arts, see [92]). The concept of reward, however, can take on different forms, as aesthetic rewards are highly abstract in nature. They are mostly culture-dependent and involve cognitive components with a critical role for learning and social influences, thus recruiting higher-order and more complex regions of the evolved brain [93,94]. 

Listeners, in their search for rewarding stimuli, may behave as biological beings who have recourse to their neural apparatus for coping with sounds. This involves the neural mechanisms for evaluation of the environment in terms of threats and dangers, but also for the search of possible benefits for survival [10,71]. The latter include, among others, the generation of affective reactions—both positive and negative—, which may be considered to have adaptive functions. Positive affect, in particular, has consequences for the expansion of cognitive and emotional resources, which are not merely subjectively felt, but which can be measured also by objective means [95]. As such, it is possible to conceive of music in terms of biologically rewarding stimuli and to link music listening with the findings from stress research—both its positive/rewarding and negative/aversive aspects—, and its connections between life experience, emotion, and health outcomes.

On the positive side, there is, first and foremost, the *empowering* impact of music—both in the short and long term—, besides its *curative* and *protective* effect. The long-term effects are the easiest to assess as they can be assessed from neuroplastic changes that occur after repeated listening or playing; the short-term effects, on the contrary, are mainly related to the benefits of having an aesthetic experience or simply enjoying the music, even for physical and mental health (see [43,96], for an overview). 

While the power of music to elicit emotional reactions and regulate the subjective emotional state is widely acknowledged, this power is highly variable across individuals with their distinct dispositional traits. For instance, in one of our previous studies [97], listening to a relaxing music track (vs. a control noise track balanced in amplitude modulations with the music) was more effective in individuals with higher scores in anxiety trait and lower scores in emotional control trait from the Big Five personality model. Several attempts have been made to link aesthetic chills to another Big Five personality trait, namely “openness to experience”—broadly characterized as comfort with novelty and motivation for cognitive exploration—[50] or to the reinstatement of social contact after separation or loss, in the case of cold chills—as a kind of thermoregulatory underpinning of social motivation [22,98]. 

In general, it has been found that a distinction can be made among listeners between *chill responders* and *non-responders* [99]. Chill responders seem to show a preference for less intensive stimuli, they like approval and positive emotional input from their environment rather than seeking thrill and adventure, and they listen to music mostly alone and separated from their surroundings rather than in social settings [46]. The neural basis of these individual differences between emotional and non-emotional responders, however, is not yet totally understood [5]. Even if it has been postulated that warm and cold chills are pursued and sought after by specific populations [46]. The whole discussion, however, can be better contextualized within the corresponding distinction between two kinds of listeners which can be classified as *empathizers* vs. *systematizers*, with the former focusing on the affective aspects of the music by identifying emotions and responding emotionally, and the latter tending to find structures and organization behind the music such as formal structures, regularities, patterns and rule systems [53,100]. 

Besides *traits* that should predispose to specific ways of listening, there seems to be also a modulation of perceptual and affective responses to music by psychological states, as exemplified in specific *listening modes*. Evidence exists, in this regard, for the role of cognitive vs. affective listening modes that can be adopted at the moment by participants during experimental sessions. In an event-related potential study by Brattico et al. [101] participants were induced by a visual prompt to switch between two listening modes when presented with 5-chord sequences ending with congruous, mildly incongruous, or highly incongruous chords: one mode was termed “cognitive” since it required the judgment of correctness of the chords, and another mode was termed “affective” since it required the judgment of the liking or disliking of the chords. While the early error-related response to the ambiguous and incongruous chords did not differ according to listening modes, later neural responses clearly differentiated the modes. Even a neural response before the chord listening started was observed indicating a mode-specific attentional resource allocation (see also [102]). 

Neuroimaging findings with fMRI confirmed the involvement of distinct neural mechanisms depending on listening modes. Liu et al. [25] asked listeners to perform three judgments of pop/rock 15-s clips in separate stimulation blocks: one involving a non-evaluative judgment (determining the gender of the singer), one involving an explicit evaluative aesthetic judgment (deciding whether they liked or did not like the clip), and one involving no judgment at all (passive listening only). Non-evaluative listening increased connectivity in the auditory-limbic brain network. In turn, the evaluative judgment strengthened intercommunication only between areas related to auditory processing and action observation, and between higher-order structures involved with visual processing.

In another fMRI study, Bogert et al. [103] modulated the visual instructions related to 4-s music clips, asking to pay attention either to the numbers of instruments playing in the clip (implicit condition) or to explicitly classify the emotions conveyed by the music (explicit condition). The implicit condition (contrasted with the explicit one) of music listening activated bilaterally the inferior parietal lobule, premotor cortex, as well as reward-related areas such as the caudate (dorsal striatum) and ventromedial frontal cortex. In contrast, dorsomedial prefrontal, and occipital areas, previously associated with emotion recognition and cognitive processing of music, were active during a listening mode focused on explicitly judging the musical emotions expressed in the clips.

In conclusion, evidence shows that aesthetic music listening, contrasted with incidental or even skillful listening, is characterized by attentional focus, namely the act of paying attention to the music with the aim to reach an evaluation [104]. This attentive internal state allows for the first initial fast reactions to sounds to become available for conscious appraisal and evaluation [25,70].

## 4. Neural Mechanisms of Coping with the Sounds

The effects of music listening can be studied from two perspectives: the psychological level of well-being and enjoyment and its underlying physiological and neurological correlates. Though it is possible to conceptually distinguish both perspectives, they are quite intertwined with the neurological level being the ultimate explaining mechanism for the generation of the aesthetic emotion of enjoyment. This is clear from recent studies within the emerging field of neuroaesthetics and neuroaesthetics of music, which try to understand the neural modulating factors that underlie aesthetic experiences in general and positive emotional responses to the music [8,58,92,105,106,107,108,109,110]. It is a challenging new field that aims at explaining musical behavior in terms of stimuli, brain physiology, and motor responses [40]. Several topics, however, are still under discussion, such as the role of neurotransmitters as dopamine in the occurrence of arousal peaks, the relation between pleasure and aesthetic reactions, the claim that all aesthetic-related reactions can be ultimately reduced to biological origins [111] and the distinction between aesthetic pleasure as a core pleasure evoked in subcortical brain processes as against conscious liking that involves also evaluating judgments which originate in higher-order cortical structures [25].

Much is to be expected here from a naturalistic approach to musical sense-making and a conception of listening in terms of “coping” with the sounds [10,112] with the aim to explore how an ordinary sensory experience can be transformed into an aesthetic experience. Two components of core affect seem to be involved in this transition, namely *arousal* and *valence*, both of which are triggered by the visceral and peripheral sensory systems. They can be defined as mental representations of bodily changes that can be valued as hedonic pleasure or displeasure with some degree of arousal [87,113] by considering the empowering impact of music, its role in arousal enhancement, and its function in stress regulation and reduction, either in a positive or negative sense. 

### 4.1. Aesthetic Listening and the Generation of Pleasure

Neuroscience has provided major insights into the positive effects of aesthetic music listening by focusing on the localization and connectivity of so-called hedonic hotspots in cortical and subcortical regions of the brain, and by examining the role of neurotransmitters in the modulation of the physical and physiological responses to the music.

The generation of pleasure—both in its sensorial and conscious aspects— depends mainly on a network of strongly connected *hedonic hotspots* within the mesolimbic pathway. They have adaptive values by helping us to want, to like, and to learn about stimuli that may possibly ensure survival, and are found along the reward circuitry in the nucleus accumbens (NAcc), the insula, the orbitofrontal cortex (OFC) and the ventral pallidum [114,115]. The subcortical hedonic hotspots are responsible for the simple and spontaneous core liking reactions to pleasurable stimuli, whereas the involvement of cortical prefrontal structures is needed for the conscious feelings of wanting or incentive salience during the appetitive phase [95,116], this network shows an interplay between more evolved neocortical areas and evolutionary older areas of the brain.

**Figure 3:**
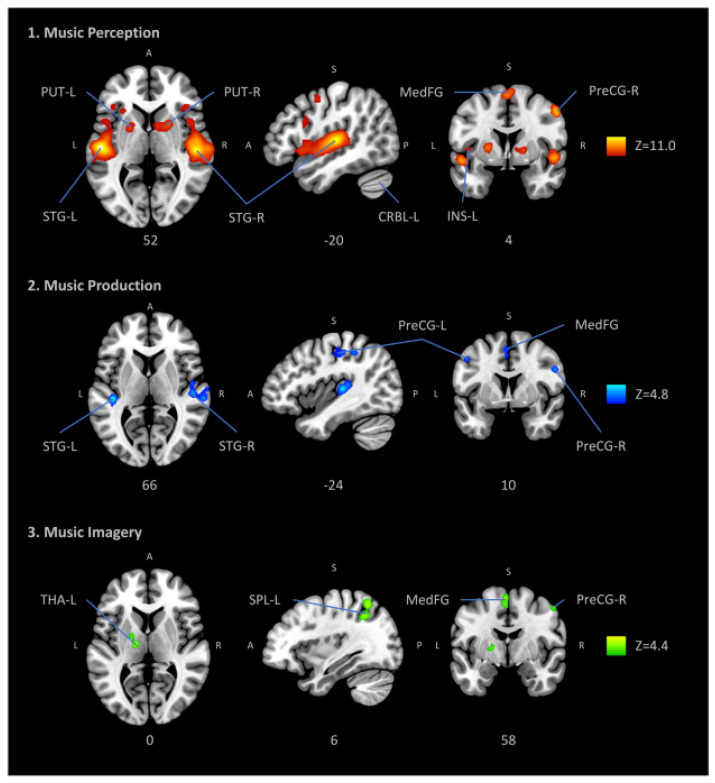
Automatic meta-analysis obtained with neurosynth.org of 163 studies utilizing the fMRI methodology and including “music” as keyword. Brain structures related to audition (supratemporal cortex), motor control (dorsomedial prefrontal cortex and cerebellum), body awareness (insula) and reward (ventral striatum) are visible. ROIs: PUT putamen, STG superior temporal gyrus (primary auditory cortex), MedFG medial frontal gyrus, CRBL cerebellum, INS insula, PreCG precentral gyrus (primary motor cortex or M1), THA thalamus, SPL superior parietal lobule, Z peak Z-value. (Figure reproduced without modification from [117]. (© Springer, Creative Commons Attribution))

Most investigations, however, have concentrated on cortical and subcortical “telencephalic sites” of aesthetic and emotional processing, such as, e.g., the dorsal and ventral striatum and amygdala, somewhat in line with a very recent meta-analysis on music listening and imagery (see Figure 3) [117]. Yet some evolutionary older levels, such as the brain stem, which houses several auditory processing mechanisms as well as core mechanisms for homeostatic regulation, have been left out to some extent, due partially to the technical difficulties of brain stem imaging, but also to the lack of theoretical frameworks for its role in hearing as related to the mechanism of homeostasis [26]. These older levels are important, however, in the sense that a distinction has been proposed between *fast* and *slow routes* of affective evaluative processes: the fast route is a primary route to the positive or negative appraisal or evaluation of stimuli, relying on quick and automatic brain responses, mostly below the level of consciousness and originating mainly in the brainstem, the primary sensory cortices, and the limbic system; the slow route, on the contrary, involves the evolutionary more evolved structures of the brain, such as the prefrontal cortex with outcomes such as the conscious liking or appraisal of a piece of art or music [118,119,120]. 

Besides these structural issues, neurochemical research has also identified the impact of *neurotransmitters* on the affective aspects of music listening. The study of combined psychophysical, neurochemical, and hemodynamic effects, in particular, may reveal peaks in the autonomic nervous system activity, which explain also the effects of music on mood (e.g., [97]. Studies with ligand-based position emission tomography—using radioligand raclopride that binds with dopamine—have shown that strong emotional responses to music lead to *dopamine release* in the mesolimbic striatal system together with sensory regions for auditory reception while listening to highly pleasurable music [93,94]. 

Some findings have been even more ground-breaking, with an unforeseen functional dissociation between the “anticipatory phase” of peak emotional experiences with dopaminergic activity in the caudate nucleus, and the “consummatory phase” of the actual experience with activity in the nucleus accumbens. This dopamine release, immediately before the climax of emotional experiences, in the caudate, is of considerable importance, as this region is highly interconnected with limbic regions such as the amygdalae, hippocampus, cingulate and ventromedial prefrontal cortex, which all mediate emotional responses. The findings point also to the direction of two distinct anatomical pathways that play different but complementary roles in the emotional experience of music, mapping somewhat onto the “wanting” and “liking” dimension of music appreciation ([121], see also [92]).

Care should be taken, however, not yet to generalize too much about the role of dopamine release. Even if it underlies both the appetitive and consummatory phases of reward, the peak of the pleasure experience and the hedonic properties or subjective pleasure that is associated with obtaining a reward may depend on the release of other neurotransmitters such as endogenous opioid peptide releases in the hedonic hotspots in the nucleus accumbens, which is a region that is implicated also in the euphoric components of psychostimulants such as, e.g., cocaine [65,115,122,123,124,125] (see also [26]). This could challenge the common view that dopamine is causally related to music-evoked pleasure through the engagement of the hedonic hotspots in favor of a milder claim that it should arise from motivational signals and cognitive appraisal so as to increase the attractiveness of the surrounding environment and to strengthen the efficacy of rewarding stimuli [125]. As such, it should intervene in the processing of diverse types of pleasures, as is the case in aesthetic experiences [65,115]. Somewhat generalizing, it can thus be stated that listening to pleasurable music is associated with activation of the NAcc and its interactions with brain structures that regulate autonomic, emotional, and cognitive functions. There is, as such, a strong link between emotional and cognitive systems which link the orbitofrontal cortex with mesocorticolimbic dopaminergic circuitry (NAcc and VTA) [65]. 

### 4.2. Music Listening, Stress, and Allostatic Load

Music, as a candidate elicitor, does not always contribute to pleasure. As a vibrational phenomenon, it may put stress on the body and the brain, it can have a role in maintaining, restoring, or even disrupting the homeostatic balance, and can, in the worst case, even become a source of allostatic load. It makes sense, therefore, to conceive of music listening in terms of “coping behavior” (see above) with a major distinction between adaptive and maladaptive ways of listening [10,126].

The concept of adaptation, first, is very useful. According to Selye—a pioneer in the study of stress—, life is a process of adaptation to the circumstances in which we exist, and health and happiness depend on the successful adjustments to the ever-changing conditions of this environmental surrounding world [127,128]. Healthy functioning, in this view, requires ongoing adjustments and alternations of the internal physiological milieu through physiological systems that exhibit fluctuating levels of activity to respond and adapt to the solicitations of the environment [129]. Stress research, however, has traditionally focused on failures in this process, emphasizing the role of *allostatic load*, which, in its etymological sense, means “stability through change”, thus emphasizing the constant dynamism of our internal physiology [130]. It can be defined as the cumulative wear and tear of the strain of physiological effects of multiple forms of adversity on several organs and tissues, due to the overactive or inefficient management of the stress responses. Allostasis, then, reflects the consequences for risk of pathology, such as organ system breakdown, compromised immune response, cardiovascular dysfunction and disease, elevated cortisol and insulin secretion, accumulation of abdominal fat, loss of bone minerals, reproductive impairments, decreased neurogenesis, increased neuronal cell death and associated atrophy in the limbic system ([131], see also [132]). 

Two major kinds of allostatic load have been identified thus far: “physiological reactivity”, as in the case of acute shifts and elevations in physiological activity in response to threatening stimuli, and “chronic elevations” beyond the basal operating ranges, operating mainly in the absence of challenging stimuli. Conditions as hypertension and diabetes are typical examples. The picture, however, is not exclusively negative as it is also possible to conceive of *optimal allostasis*. It is a challenging approach that has marked a kind of paradigm shift in stress research by focusing not exclusively on failures in the adaptation process [129]. An operational approach to such optimal allostasis, therefore, should include not only the maintenance of load indicators in normal operating ranges but also the measurement of selected brain opioids such as β-Endorphins and leucine and methionine enkephalins, which have powerful effects in counteracting negative emotions and favoring positive ones [133,134]. The release of dopamine from the catecholamine systems, the central nervous system opioid peptides, and oxytocin are also of particular importance in this regard (see [129] for an overview). 

The effects of allostatic load, further, are reflected in biological stress responses, such as the neuroendocrine, autonomic, and immune system responses, which put high strains on the mobilization from the *hypothalamic-pituitary-adrenal axis* [65], with a corresponding cascade of hormone secretions, going from the hypothalamic-driven release of corticotropin-releasing factor (CRF), to the pituitary-driven release of corticotropin to the adrenal-driven release of cortisol [129,135]. Activation of the sympathetic nervous system by stress-inducing stimuli, further, results in the release of catecholamines such as norepinephrine and epinephrine, driven by the medulla of the adrenal gland. The immune function, finally, can be compromised by the workings of these hormones and transmitters. 

The above-described neurotransmitter-mediated neural activity within the reward pathways, on the other hand, regulates emotions and mood through changes in autonomic and physiological responses during music listening. This often leads to a relaxed smoothened state through psychological processes of decentering and non-attachment, so as to decouple the “sensory” and “affective” components of stressors with a resulting reduction of the sympathetic tone and suppression of mobilization by the hypothalamic-pituitary-adrenal axis. The aim of such decentering is to decrease stress responses to innocuous cues and to foster a rapid return to physiological and emotional baseline situations in response to real threats. Such reductions should then be visible across physiological mediators such as the adrenomedullary catecholamines (epinephrine and norepinephrine), adrenocortical glucocorticoids (cortisol), pituitary hormones (ACTH, prolactin, and growth hormones), and cytokines from cells of the immune system (IL-1, IL-6, and TNF-α) [136]. 

Hence, music can have a mediating role in the interactions between these physiological organ systems with a possible regulation of systemic stress hormone levels. Two markers of the HPA axis—ß-endorphin and cortisol—, have been found to decrease as the result of engaging with music, but not all findings point in the direction of lowering levels of activation. Stimulating music, e.g., can induce increased levels of plasma cortisol, ACTH, prolactin, growth hormone, and norepinephrine [65].

Generalizing a little, it can be stated that music can regulate stress, arousal, and emotions by initiating reflexive brainstem-mediated responses, which include heart rate, blood pressure, skin conductance, and muscle tension [137]. A distinction should be made, however, between those levels of stress that are perceived as harmful or annoying and those that are experienced as being beneficial for better coping behavior. It is a conception that is related to the psychobiological model of arousal, which states that enjoyment is optimal at intermediate arousal levels. 

Applied to music, this means that it makes sense to attune ourselves to sound environments and sonic landscapes, including music, that provides stimulation in the optimal arousal zone [10,138,139], allowing us to cultivate positive adaptive reactions to beneficial stressors as well as to avoid possible distress triggered by harmful stimuli. It brings us to Selye’s concept of *eustress*, which he contrasted with *distress*, as the syndrome that is triggered by unspecific harmful stimuli or activities [127,128,140]. Eustress represents the pleasant stress of fulfillment, including both the properties of the stressor, considered to be beneficial in that case, the effort that is valued in terms of positive valence, and the effects that guarantee no damaging outcomes. Stressors, then, can be considered beneficial when they do not exceed the capacity for the maintenance or restoration of homeostasis [141].

Both negative-aversive and positive-rewarding stress, however, is associated with increased activation of the HPA axis. It means, finally, that aesthetic engagement with music reveals a unique neural architecture that connects physiological responses typical of stress to the combination of positive and negative affect, which leads ultimately to an expansion of our cognitive-emotional states. This is exemplified most typically in the experience of awe and aesthetic chills, which are characterized by vastness and the need for accommodation [142,143].

## 5. Conclusions

In this paper, we have focused on how certain individual, cultural, and acoustic factors affect music listening with a special focus on their neurobiological underpinnings, somewhat in line with a very recent meta-analysis on music listening and imagery (see Figure 3), which conceives of music mainly as a sensorimotor and cognitive process, but leaving out most of the affective and even more the aesthetic aspects of the experience, such as judgment and eudaimonia [117]. Starting from recent developments for the acoustic description of music and ways of listening, we have elaborated on the possible effects of music, which may be modulated or even biased considerably by the personality and learning history of each individual listener. By stressing the role of ontogenetic development, we have pointed out the malleability and learnability of listening as a skill. A distinction should be made, however, between skillful listening, as exemplified in expert listeners with formal musical training, and aesthetic listening, which adds some additional level of musical engagement to this level of sophistication. As such, the musical-aesthetic experience is considered a privileged and target example of musical engagement, in the sense that it combines empathic/emotional listening with aesthetic valuing and high-level cognitive processing. In neurological terms, this combines both cortical and subcortical processing of the musical stimuli with major connections between three regions, namely the auditory cortex, the prefrontal cortex involved in moral judgment, and the limbic and paralimbic regions of the emotional brain. It has been argued, moreover, that continuous and ongoing engagement with music with an aesthetic attitude can induce neuroplastic changes, both in the short and long term, with structural and functional modification of the brain as a possible outcome. The picture that emerges, finally, is a new approach to music listening that ties together arguments from distinct disciplines, but which all converge in the direction of a comprehensive aesthetic approach that maneuvers elegantly between biological and cultural perspectives (see also [19,20,21]). The focus of this paper, however, was on the former rather than the latter. Yet it opens up additional perspectives for future research.
